# Unusual rupture of left ventricular pseudo-false aneurysm secondary to subacute anterolateral myocardial infarction: a case report

**DOI:** 10.1186/s13019-019-0915-x

**Published:** 2019-05-17

**Authors:** Masaho Okada, Hirotaka Watanuki, Kayo Sugiyama, Yasuhiro Futamura, Katsuhiko Matsuyama

**Affiliations:** Department of Cardiac Surgery, Aichi Medical University, 1-1 Yazakokarimata, Nagakute, Aichi Japan

**Keywords:** Left ventricular pseudo-false aneurysm, Subacute cardiac rupture, Intramyocardial dissecting hematoma

## Abstract

**Background:**

Left ventricular (LV) pseudo-false aneurysm is a rare complication secondary to myocardial infarction and is caused by intramyocardial dissecting hematoma due to fragile myocardium. Very occasionally, intramyocardial dissecting hematoma appears as a neocavitation entirely contained within the myocardial wall (so called “pseudo-false LV”) and is an unusual form of subacute cardiac rupture.

**Case presentation:**

A 38-year-old male experienced chest discomfort 3 weeks ago, which improved within few days. However, after that episode, he presented at our hospital with rapidly deteriorating severe breathlessness in a preshock state with acute heart failure. Emergency coronary angiography revealed an occluded left anterior descending artery. An intra-aortic balloon catheter was inserted because of unstable hemodynamics. Enhanced computed tomography revealed extensive aneurysm formation in the LV anterior wall and contrast leakage from the inner cavity to the LV myocardium, with a moderately accumulated pericardial effusion. Emergency surgery revealed a large aneurysmal sac on the anterior wall, slightly attached to the pericardium. A 5-mm, slit-like, oozing-type, rupture site was detected in the LV after dissecting the pericardium.

**Conclusions:**

To our knowledge, this is the first report of a pseudo-false aneurysm on the LV anterior wall. Subacute rupture of pseudo-false LV aneurysm is rare.

## Background

A cardiac free wall rupture following acute myocardial infarction is a rare and fatal condition. Frequently, an intramyocardial dissecting hematoma may appear as a neocavitation entirely within the myocardial wall [1]. This unusual form of subacute cardiac rupture is termed “left ventricular (LV) pseudo-false aneurysm” and is characterized by the development of dissection planes between the spiral muscles of the ventricle [2]. We report an uncommon case of ruptured LV pseudo-false aneurysm following subacute anterior myocardial infarction.

## Case presentation

**Fig. 1:**
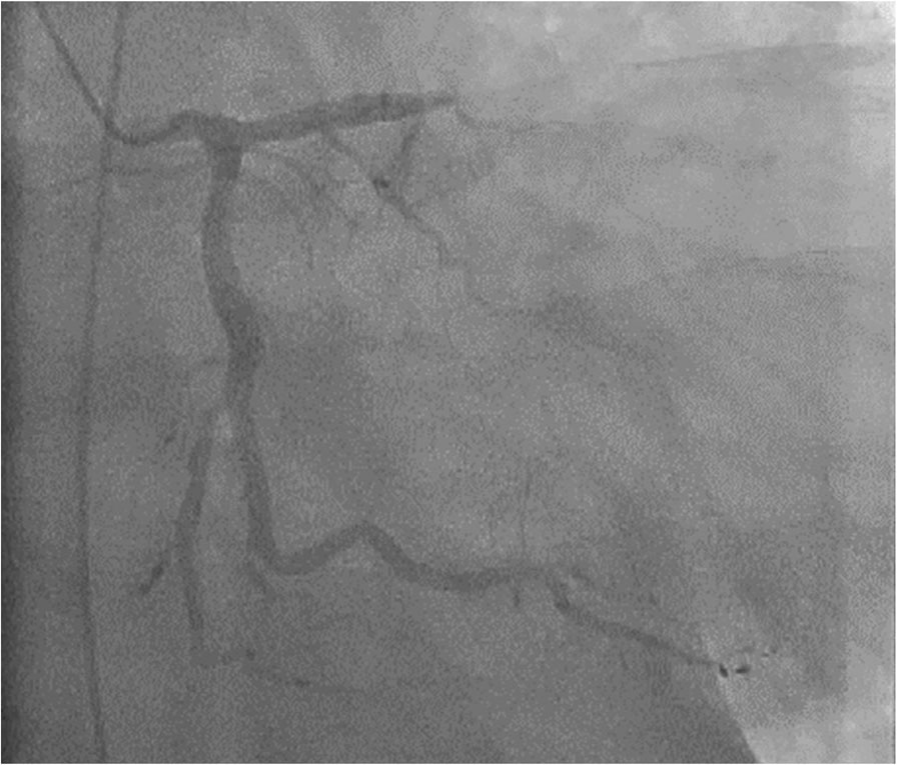
Preoperative coronary angiography. Occluded LAD artery is in the middle portion

**Fig. 2:**
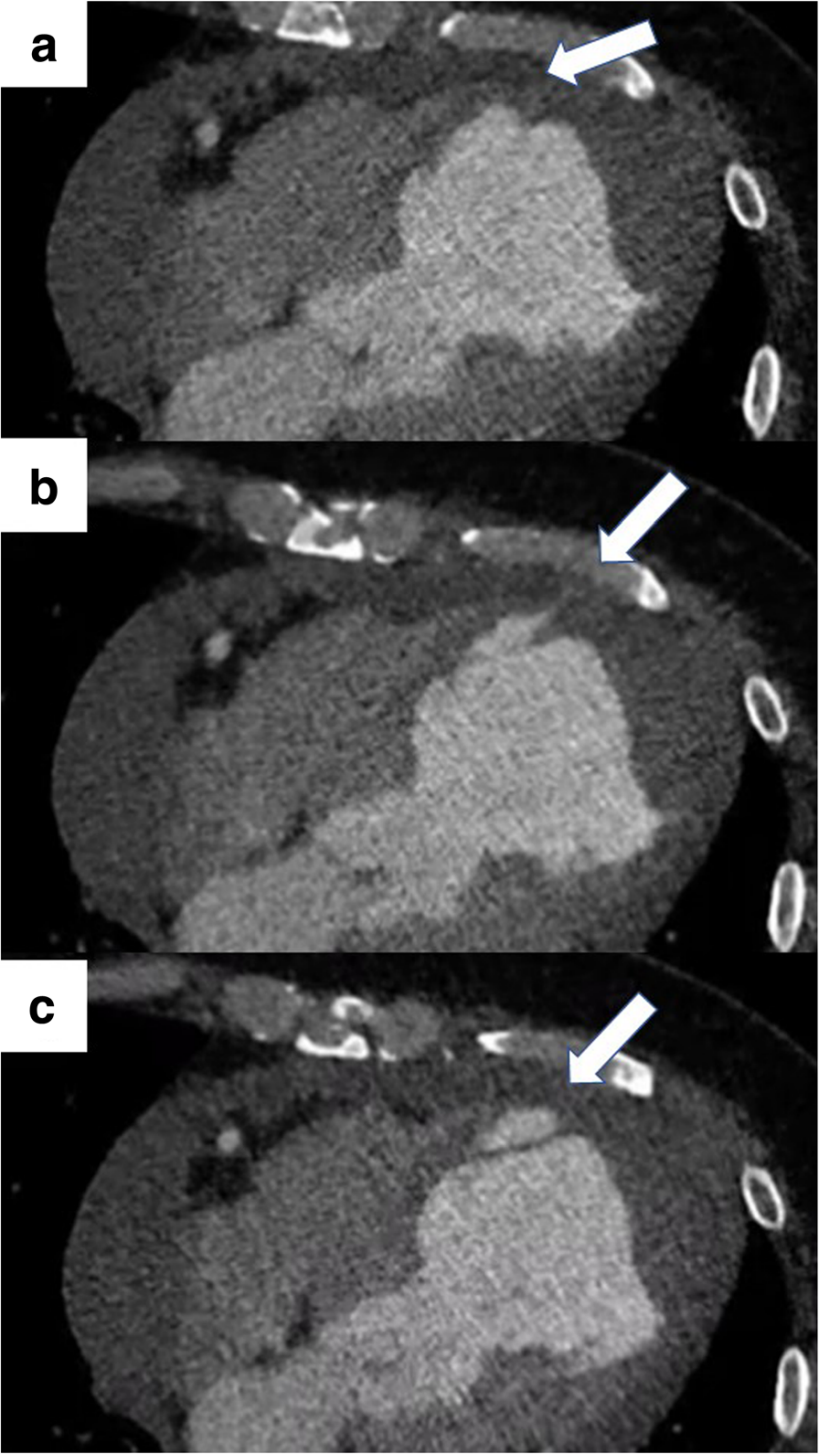
Preoperative computed tomography. (**a**–**c**) Extensive aneurysm formation on the anterior LV wall, with pericardial effusion accumulation. (**b**) The aneurysm showing leakage of contrast from the inner cavity to the LV myocardium. (**c**) Intra-LV wall aneurysm with the dissected myocardium

**Fig. 3:**
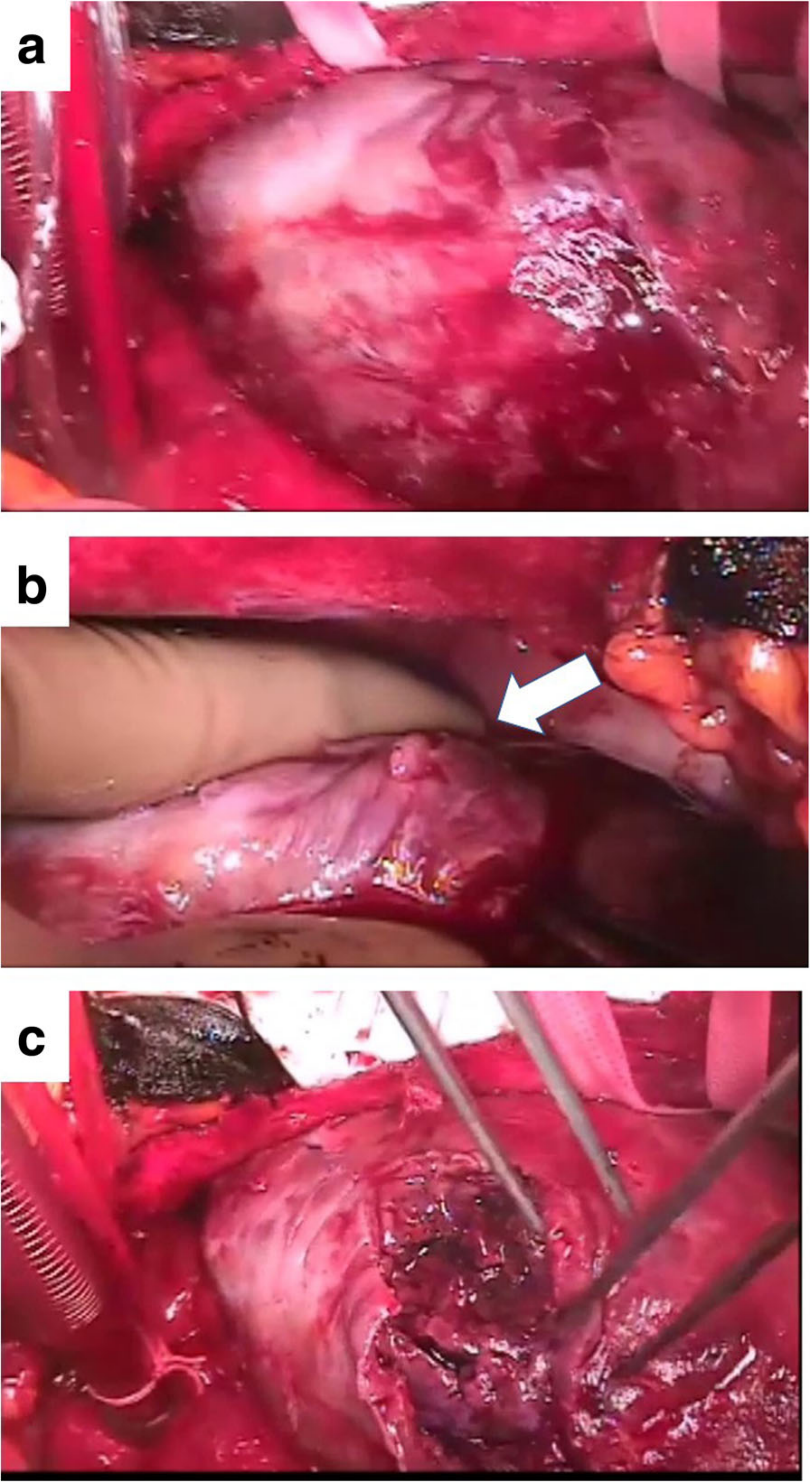
Intraoperative findings. (**a**) A large aneurysmal formation on the anterior LV wall, slightly attached to the pericardium is noted. (**b**) After dissecting the pericardium, a 5-mm, slit-like LV rupture site is found in the aneurysm, which caused cardiac tamponade. (**c**) The anterior myocardium of the aneurysm containing intramyocardial heavy and flesh hematoma

**Fig. 4:**
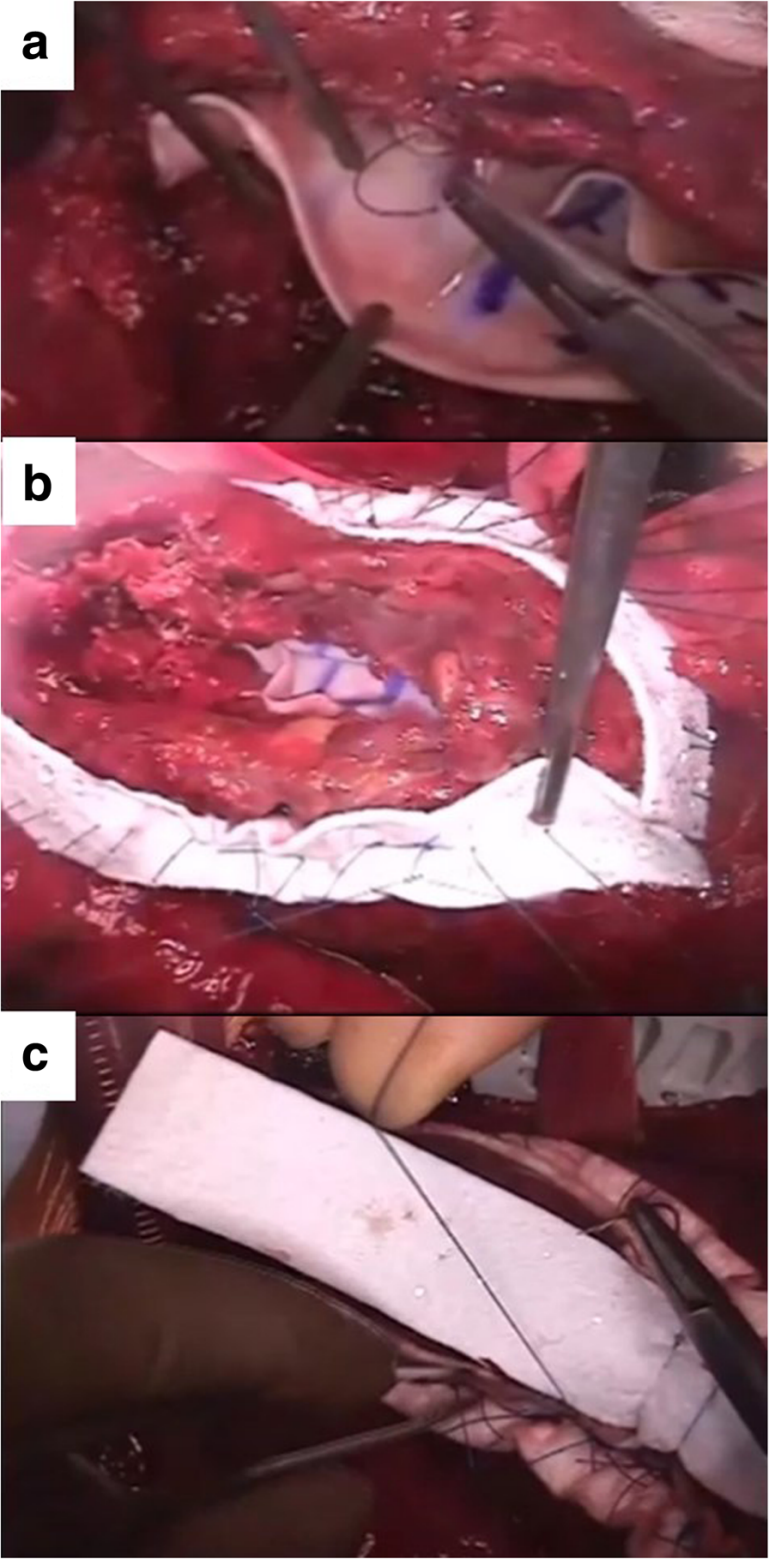
Surgical procedure for reconstruction of LV wall (**a**) The LV defect was closed using the patch with transmural interrupted mattress sutures to avoid excessive reduction in the ventricular volume. (**b**) The ventricular edge was closed by interrupted sutures using two Teflon felt strips to reinforce the suture from the outside. (**c**) A second running suture was used to ensure a secure left ventriculotomy closure placing another Teflon felt strip in the middle of the edge

**Fig. 5:**
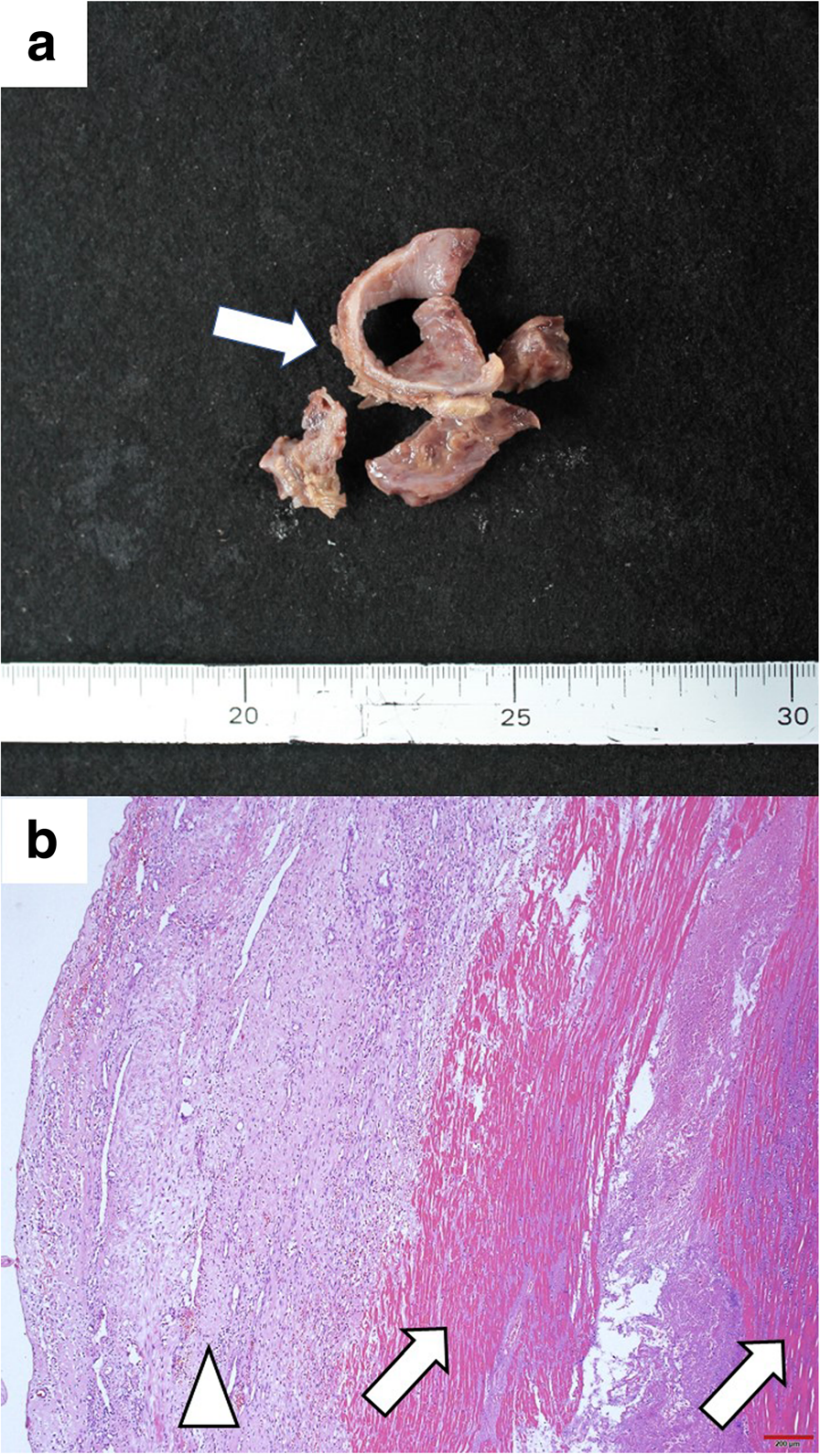
Pathological findings of the LV aneurysmal rupture site (**a**) The resected LV aneurysmal rupture site specimen. (**b**) Pathological finding in the ruptured myocardium (low power field, × 4). The myocardium of the left ventricular wall shows myocardial necrosis (*arrows*), and the surrounding tissue shows cell infiltration. In particular, there is granulation tissue formation at the epicardium side (*arrowhead*)

A 38-year-old man (height, 172 cm; body weight, 120 kg; body mass index, 37) experienced chest discomfort 3 weeks ago, which improved within few days. However, after that episode, he was admitted with rapidly deteriorating severe breathlessness in a preshock state with acute heart failure. The patient had a smoking habit and hyperlipidemia. Electrocardiography revealed abnormal Q waves and slight ST elevation in the aVl, V1, V2, and V3 leads. However, laboratory findings demonstrated creatine kinase (CK) and CK-MB levels within the normal range. Echocardiography revealed aneurysmal enlargement in the anterior wall and moderate-to-massive pericardial effusion, and severely reduced wall motion of LV. Emergency coronary angiography demonstrated an occluded left anterior descending artery (LAD; Fig. 1). Circulatory support with an intra-aortic balloon pumping (IABP) catheter was started because of unstable hemodynamics. Enhanced computed tomography showed extensive aneurysm formation on the anterior LV wall and contrast from the inner cavity to the LV myocardium, with moderately accumulated pericardial effusion (Fig. 2a–c). Emergency surgery was performed, and his blood pressure ranged 80 to 90 / 50 to 60 mmHg, with 40 mmHg for PA and 20 mmHg for CVP. After the median sternotomy, bloody pericardial effusion (400 ml) was drained, and cardiac tamponade was relieved. A large aneurysmal formation was noted on the anterior LV wall, slightly attached to the pericardium (Fig. 3a). Cardiopulmonary bypass (CPB) was established with an ascending aorta and bicaval cannulation. After dissecting the pericardium, a 5-mm, slit-like LV rupture site was found in the aneurysm, which caused cardiac tamponade (Fig. 3b). Following cardiac arrest by antegrade cardioplegia, the middle aneurysm portion was dissected parallel to the LAD. The anterior myocardium comprised intramyocardial heavy and flesh hematoma and necrotic myocardium (Fig. 3c). Of note, the anterior and posterior papillary muscles were not involved. After removing the hematoma and debriding the necrotic tissue, the anterior wall defect measured 10 × 7 cm. Traction sutures were placed at each anticipated closing line. Then, two sheets of bovine pericardial patch were tailored to the anterior wall defect shape, which was 5 cm × 10 cm. The LV defect was closed using the patch with transmural interrupted mattress sutures to avoid excessive reduction in the ventricular volume (Fig. 4a). Subsequently, the ventricular edge was closed with interrupted sutures using two Teflon felt strips to reinforce the suture from the outside (Fig. 4b). A second running suture was used to ensure a secure left ventriculotomy closure, and another Teflon felt strip was placed in the middle of the edge (Fig. 4c). Cardiopulmonary bypass was easily weaned. He was extubated the following day, and the IABP was smoothly removed. Postoperative echocardiography revealed an improvement in LV function (LVEF:40%), without mitral regurgitation. Postoperative cardiac magnetic resonance image revealed a well-reconstructed LV. He was discharged without any complication 3 weeks postoperatively. The LV aneurysmal rupture site specimen was sent for pathological study (Fig. 5a). Pathological findings showed myocardial necrotic tissue with cellular infiltration within the aneurysmal wall, consistent with a pseudo-false aneurysm, which was contained by the elements of the ventricular wall (Fig. 5b).

## Discussion and conclusions

In 1981, Stewart et al. [3] reported the first case of an LV pseudo-false aneurysm, which is a rare complication secondary to myocardial infarction. Although this condition is caused by intramyocardial dissecting hematoma due to fragile myocardium, the hematoma does not dissect completely through to the epicardium contained within the infarct myocardial area as opposed to a pseudoaneurysm. The differential diagnosis between pseudoaneurysm and pseudo-false aneurysm is definitively reached through pathophysiological examination. In the present case, the pathological findings of the LV wall containing the myocardial tissue were consistent with those of a pseudo-false aneurysm, which is contained by the elements of the ventricular wall. Also, intramyocardial hematoma was considered to suddenly develop in the subacute phase of anterior myocardial infarction [4]. Fortunately, bleeding from the ruptured dissecting myocardium was minimally controlled because of slight attachment to the epicardium.

In the majority of reported cases, pseudo-false aneurysms develop in the posterior or inferior wall [5]. Otherwise, rupture to right ventricular is also more common [6, 7]. However, a pseudo-false aneurysm occurring in the anterior wall, as observed in the present case, are extremely rare and has not been reported previously to our knowledge. Anatomically, this may be attributed to the crossing of two ventricular myocardial bands at the anterior wall, producing two helicoid spirals [8].

The choice of repair for a ventricular aneurysm depends on the condition of the myocardium. In the acute or subacute phase, the LV defect was closed using a bovine pericardial patch and transmural interrupted mattress sutures because of the fragile myocardium. In addition, Teflon pledgets were used to reinforce the suture on the outside. Subsequently, the ventricular edge was closed with interrupted and running sutures using Teflon felt strips.

To our knowledge, this is the first report of an LV anterior wall pseudo-false aneurysm. Subacute rupture of pseudo-false LV aneurysm is rare.

## Abbreviations

CK: Creatine kinase
IABP: Intra-aortic balloon pumping
LAD: Left anterior descending artery
LV: Left ventricular
